# Afforestation-Induced Shifts in Soil Bacterial Diversity and Community Structure in the Saihanba Region

**DOI:** 10.3390/microorganisms12030479

**Published:** 2024-02-27

**Authors:** Kai-Chuan Huang, Wen Zhao, Jun-Ning Li, Reyila Mumin, Chang-Ge Song, Hao Wang, Yi-Fei Sun, Bao-Kai Cui

**Affiliations:** State Key Laboratory of Efficient Production of Forest Resources, School of Ecology and Nature Conservation, Beijing Forestry University, Beijing 100083, China; huangkc1014@bjfu.edu.cn (K.-C.H.); zhaowendrlw@163.com (W.Z.); lijunning17@mails.ucas.ac.cn (J.-N.L.); ramilla@163.com (R.M.); changgesong@126.com (C.-G.S.); 15663733531@163.com (H.W.)

**Keywords:** Saihanba Mechanized Forest Farm, forest restoration, bacterial community structure, functional prediction, co-occurrence network

## Abstract

Afforestation plays a pivotal role in ecosystem restoration, exemplified by the Saihanba Mechanized Forest Farm, the world’s largest planted forest; however, the assembly mechanisms and interactions of soil microbial communities in such forests remain inadequately understood. This study aimed to elucidate the impact of different afforestation tree species, namely *Larix gmelinii* var. *principis*-*rupprechtii*, *Picea asperata*, and *Pinus sylvestris* var. *mongolica*, on soil bacterial diversity and community structure in comparison to grassland. Sixty soil samples were collected at a 20 cm depth, and high-throughput sequencing was employed to identify bacterial communities and assess their interactions with environmental factors. A total of 6528 operational taxonomic units (OTUs) were identified, with *Solirubrobacter*, *Conexibacter*, *Bacillus*, *Massilia*, *Gaiella*, *Acidibacter*, and *Vicinamibacter* being the dominant genera. Afforestation significantly impacted soil bacterial alpha diversity, with notable influence from key soil chemical properties, including available phosphorus (AP), cation exchange capacity (CEC), and the carbon-to-nitrogen ratio of soil organic matter (SOM-C/N). The Mantel test highlighted pH, the Normalized Difference Vegetation Index (NDVI), and spatial variable (dbMEM) as primary environmental factors influencing dominant bacterial genera. The bacterial community structure demonstrated deterministic homogeneous selection, wherein SOM-C/N emerged as a significant factor influencing the dissimilarity of soil bacterial communities. Furthermore, plantation soils exhibited a more complex network structure than grassland soil, highlighting the crucial role of bacterial communities in vegetation changes and providing valuable insights into their response to environmental factors during the reforestation process.

## 1. Introduction

Afforestation, a crucial method for vegetation restoration, plays a vital role in enhancing soil quality, mitigating desertification, and establishing windbreaks [1,2]. The proliferation of artificial vegetation significantly alters soil environmental conditions, impacting the composition and diversity of soil microorganisms [3,4], which, in turn, are integral to soil material cycling [5,6]. During afforestation, factors like tree species, vegetation type, soil texture, and climate conditions influence soil microorganism diversity and community composition [7,8]. Yet, there is a lack of consensus on the direction of changes in soil microbial structure and diversity post-silviculture and their impact on system stability [9,10]. Moreover, limited studies on the response mechanisms of soil microorganisms, particularly bacteria, to different silvicultural tree species and soil environmental changes impede a comprehensive understanding of soil bacteria and their ecological functions.

Changes in soil bacterial community structure and diversity can influence the diversity of above-ground vegetation and biogeochemical cycles, which are critical indicators of ecosystem recovery following afforestation [11,12]. Different forest types demonstrate significantly varied capacities to buffer soil acidification [13]. The effects of afforestation on bacterial community structure and function are modulated by shifts in soil chemistry. Bacterial communities rely on nutrient availability and strongly interact with the levels of soil carbon, nitrogen, and phosphorus [14,15,16]. Furthermore, variations in bacterial community composition impact the soil carbon cycle and shape interspecific interactions, thereby controlling secondary metabolites and nutrient structure in the soil environment [17,18,19]. The structure of bacterial networks reflects soil ecosystem stability, with key species playing a vital role in biogeochemical cycle regulation and plant nutrient acquisition [20,21]. Unveiling co-occurrence networks and key species in the community can enhance our understanding of community assembly processes, providing microbiological support for sustainable forest development [22,23]. Despite this, soil microbial community composition and diversity are often overlooked in assessing the ecological effects of existing silvicultural models, necessitating a detailed exploration of soil bacterial community responses to afforestation for a more comprehensive understanding of plantation ecosystem stability and their responses to environmental changes.

The Saihanba Mechanized Forest Farm, situated in the northern area of Hebei Province, China, stands as the world’s largest plantation forest, established in the 1950s. While our previous study comprehensively examined soil fungi in the Saihanba region, analyzing composition, functional variances, and underlying factors influencing fungal communities across various vegetation types [24], there is a significant gap in understanding soil bacteria within this region. We selected the main afforestation species in the forest site, that is, *Larix gmelinii* var. *principis*-*rupprechtii* (LF), *Picea asperata* (SF) and *Pinus sylvestris* var. *mongolica* (PF), and compared them with grassland (GL). Combined with high-throughput sequencing technology, we tried to answer the following questions: (1) How did the bacterial diversity, community structure and functional groups change after afforestation? (2) Does afforestation change the assembly process of bacterial communities? and (3) What are the driving factors for these changes or constraints?

## 2. Materials and Methods

### 2.1. Study Area and Soil Sampling

The Saihanba Mechanized Forest Farm (116°51′–117°39′ E, 41°02′–42°36′ N, 1400 m a.s.l.) is situated on the southern edge of the Inner Mongolia Plateau, at the forefront of the Hunshandake Sandy Land. The region features aeolian, meadow, and boggy soils, characterized by a continental monsoon climate with an average annual temperature of −1.4 °C and precipitation of 450.1 mm [25]. Saihanba is connected with the mountains in the north of Hebei Province. The upper boundary of the landform is between the plateau and the mountains, and it belongs to the forest-grassland transition zone. The soils are predominantly sandy. According to the terrain classification, the area is divided into two sections: the upper section consists of aeolian sand soil, while the lower section comprises mountainous brown soil. Before the establishment of Saihanba Mechanized Forest Farm, it was a plateau and barren hilly landform. Since the establishment of the site in 1962, 62,000 hectares of artificial forests have been constructed. The artificial forests are mainly *Larix gmelinii* var. *principis*-*rupprechtii*, *Picea asperata*, and *Pinus sylvestris* var. *mongolica*. After over 60 years of afforestation efforts, the forest cover has remarkably increased from 11.4% to 82%.

**Figure 1:**
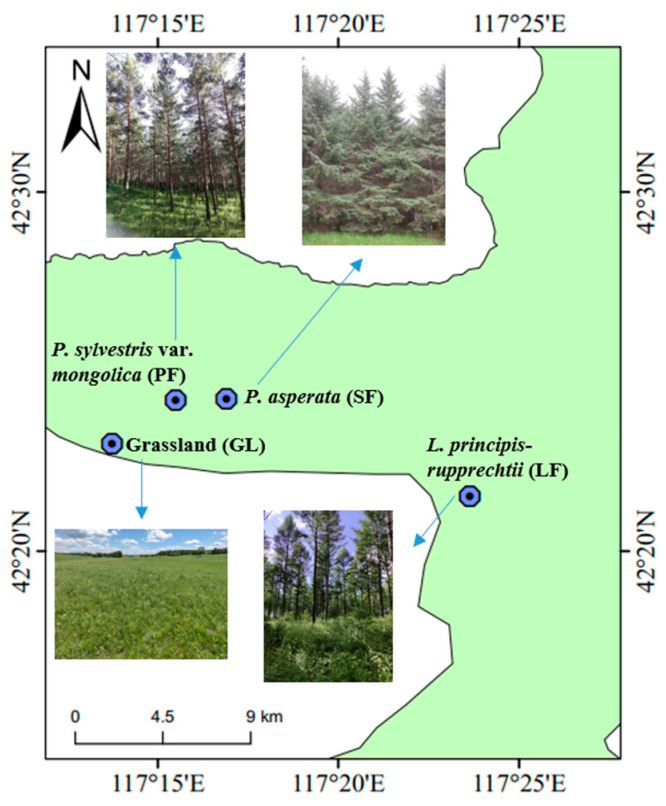
Geographical location map of sampling sites. The sampling sites are located in Saihanba Mechanized Forest Farm, Hebei Province, China.

In July 2022, the soil samples were collected from four major vegetation types, including GL, LF, SF and PF (Figure 1; Table 1). The planted forests are mainly pure forests, with the majority of grasses, composites, and ferns among the understory herbaceous plants, and few vines. Trees representative of each quadrat were selected for age determination using an increment borer, while their diameters at breast height (DBH, 1.3 m from the ground) were measured using a circumference ruler. Fifteen sampling plots (20 m × 20 m) were established in each vegetation type separately, and a total of 60 samples (4 vegetation × 15 samples) were collected. Nine random soil cores (20 cm in depth) were collected from each sampling plot using a soil auger (5 cm in diameter), which were passed through a 2 mm sieve and then mixed to form a composite sample. A portion of the encapsulated samples were stored in an insulated box filled with dry ice for rapid transport to the laboratory, then stored at −80 °C for DNA extraction. Another part of the sample was air-dried at room temperature and assessed using soil agrochemical analysis [26] and the experimental method outlined by Guo et al. [27]. In brief, soil pH was measured utilizing the acidimeter method, soil organic carbon (SOC) was assessed using the potassium dichromate volumetric method, soil total nitrogen (TN) was determined via the Kjeldahl method, soil available phosphorus (AP) was quantified using the molybdenum antimony colorimetric method, soil cation exchange capacity (CEC) was evaluated employing the ammonium acetate exchange Kjeldahl method, and SOM-C/N represented the ratio of SOC to TN.

### 2.2. DNA Extraction and Bioinformatic Analysis

Total soil DNA was extracted using the DNeasy Power Soil Pro Kit (QIAGEN, Frankfurt, Germany), with approximately 0.5 g of fresh soil per sample, following the kit’s extraction protocol. The purity and integrity of the extracted DNA were assessed through 1.2% agarose gel electrophoresis, while the concentration and purity were verified using NanoDrop ONE (Thermo Fisher Scientific, Waltham, MA, USA). The amplification of the V3–V4 region of the bacterial 16S rRNA gene employed primers 338F (ACTCCTACGGGAGGCAGCA) and 806R (GGACTACHVGGGTWTCTAAT) in a 25 μL system. Purification and quantification procedures adhered to the protocol outlined by Wang et al. [28]. For purification and recovery, magnetic beads (Vazyme VAHTSTM DNA Clean Beads) were added at 0.8 times the volume of the PCR product. The recovered PCR-amplified products underwent fluorescence quantification using the Quant-iT PicoGreen dsDNA Assay Kit (Thermo Fisher Scientific, Waltham, MA, USA), with measurements conducted on a Microplate reader (BioTek, FLx800, Burlington, VT, USA). Equimolar concentrations of all samples were pooled and subjected to paired-end sequencing on the Illumina MiSeq platform by Personal Biotechnology Co., Ltd. (Shanghai, China). Raw sequences were processed using the QIIME2 platform (version 2021.2) [29,30]. The qiime cutadapt plugin was employed to eliminate sequence primers, followed by de-duplication, quality control, merging, and chimera removal using the qiime dada2 plug-in [31]. Operational taxonomic units (OTUs) with a 97% similarity were obtained using the qiime vsearch plugin. The qiime feature-classifier plugin was utilized to classify and annotate OTUs against the SILVA reference database [32]. The raw sequencing data have been uploaded to the NCBI database with the accession number PRJNA967474.

### 2.3. Statistical Analyses

Statistical analyses of OTU and annotated information tables were conducted in R (version 4.1.1). The “SRS” function was utilized to rarefy each sample to the lowest number of sequences (11792). Calculation of the alpha diversity index employed the MicrobiotaProcess package (version 1.6.6) [33]. Beta diversity among different samples was evaluated based on the Bray–Curtis distance, and principal coordinate analysis (PCoA) was executed using the vegan package [34]. A distance-based Moran’s eigenvector map (dbMEM) was constructed from the latitude and longitude coordinates of the samples through the dbmem function in the adespatial package. The Normalized Difference Vegetation Index (NDVI) of the above-ground vegetation of each sample was extracted using ENVI (version 5.3).

Community assembly was assessed using the NST and geosphere packages [35]. The pNST function compares the phylogenetically weighted abundance beta mean nearest taxon distance (β-MNTD) with the null model, quantifying the deviation using the beta nearest taxon index (β-NTI). A |β-NTI| > 2 indicates a significant deviation from the null model’s predicted value, signifying either higher or lower turnover rates of the actual community phylogeny than expected. This suggests deterministic community assembly processes. Conversely, a |β-NTI| < 2 indicates conformity to the null model’s prediction range, suggesting stochastic community assembly processes. Additionally, β-NTI < −2 indicates homogeneous selection dominance, while β-NTI > 2 indicates variable selection (or heterogeneous selection) dominance. When |βNTI| < 2, it implies that community construction is influenced by stochastic processes such as homogenizing dispersal, dispersal limitation, and undominated processes.

Stepwise multiple regression analysis of microbial diversity and environmental factors was conducted using the stepAIC function, and variance decomposition and hierarchical classification were performed using the rdacca.hp function [36]. The redundancy analysis (RDA) of microbial community composition with environmental factors was executed using the vegan package. The function of the bacterial community was determined using Functional Annotation of Prokaryotic Taxa (FAPROTAX) [37]. Spearman correlations between OTUs (relative abundance > 0.01%) were calculated using the WGCNA package for co-occurrence network analysis (with selection criteria of *R* > |0.6| and *p* < 0.05). Visualization was carried out in a Fruchterman–Reingold layout using Gephi (https://gephi.org/; accessed on 12 September 2023). Plotting was performed using Origin 2022b software (OriginLab Corporation, Northampton, MA, USA) and ggplot2 package (version 3.4.2) [38]. ANOVA and non-parametric tests were conducted using SPSS (version 27.0; IBM Corporation, Armonk, NY, USA), with *p* < 0.05 considered statistically significant.

## 3. Results

### 3.1. Soil Chemical Properties across Four Vegetation Types

Substantial variations in soil chemical properties were observed among the four vegetation types in the Saihanba area (Table 2). Soil pH exhibited a significant elevation in GL and PF compared to LF and SF, whereas AP demonstrated a significant increase in LF and SF compared to GL and PF. LF soils exhibited the highest levels of SOC, TN, and CEC, while PF displayed the lowest TN and CEC. The values of SOM-C/N demonstrated a significant increase from GL to PF.

### 3.2. Bacterial Community Diversity and Structure across Four Vegetation Types

**Figure 2:**
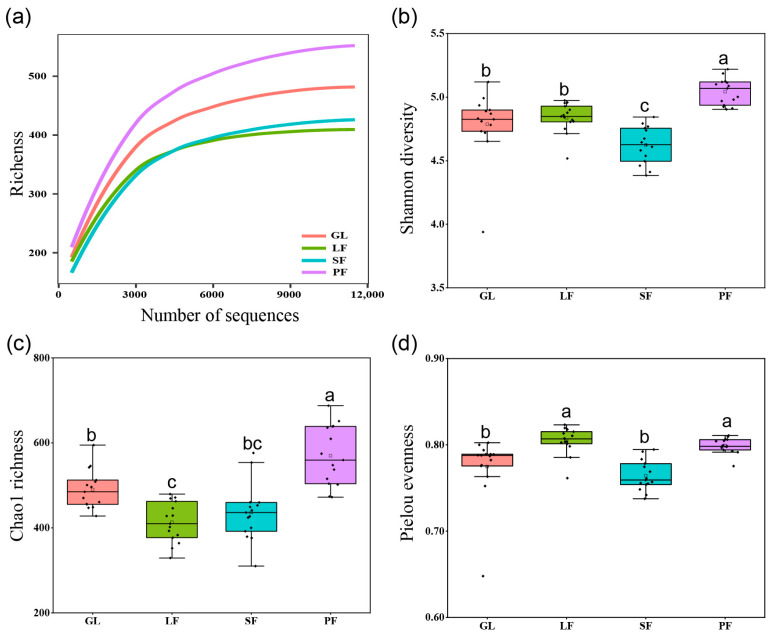
Alpha diversity of bacterial community across four vegetation types ((**a**): rarefaction curve; (**b**): Shannon index; (**c**): chao1 index; (**d**): Pielou index). Different lowercase letters indicate significant differences among the four vegetation types at *p* < 0.05 based on Tukey’s HSD.

Significant variations in soil bacterial diversity were observed across different sites in the Saihanba area (Figure 2). Rarefaction curves showed the number of OTUs at increasing sequence depths of samples, indicating that our study captured most bacterial members from each vegetation type (Figure 2a). PF exhibited the highest Shannon and Chao1 indices, while SF had the lowest Shannon index. The Pielou evenness index of soil bacteria in PF and LF significantly surpassed that in SF and GL.

**Figure 3:**
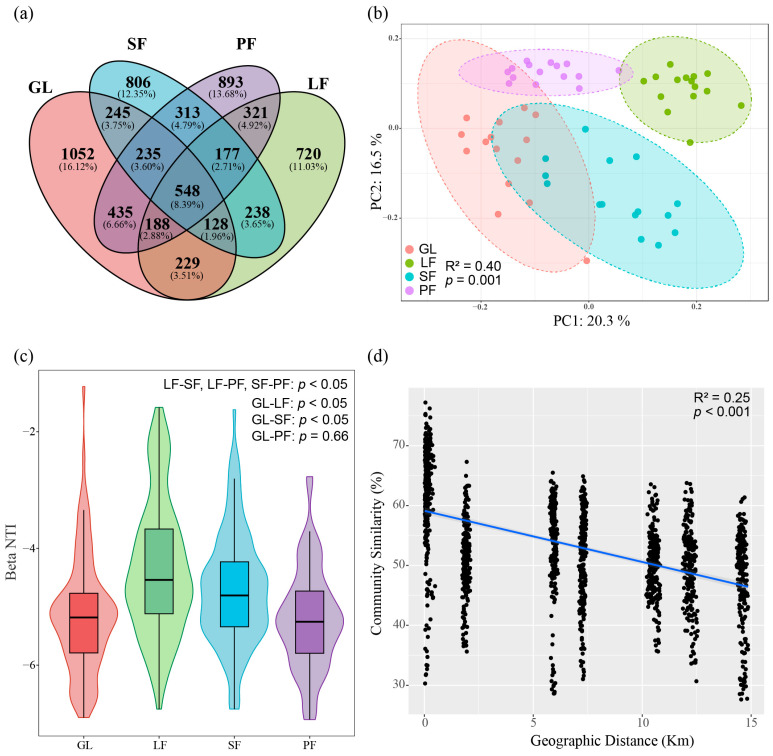
Structural differences of bacterial community across four vegetation types ((**a**): Venn diagram; (**b**): principal coordinate analysis; (**c**): distribution of Beta Nearest Taxon Index (βNTI); (**d**): distance–decay relationship, the blue line represents least squares linear regression, with the regression coefficients tested using a *t*-test (R^2^ = 0.25, *p* < 0.001)).

The Venn diagram (Figure 3a) illustrated 548 common OTUs across the four vegetation types, with unique OTUs ranging from 720 (LF) to 1052 (GL). PCoA at the OTU level, based on Bray–Curtis distance (Figure 3b), revealed that PC1 and PC2 explained 20.3% and 16.5% of the total variation, respectively. The soil bacterial community structure of LF was distinctly separated from other vegetation types (PERMANOVA, *R*^2^ = 0.40, *p* = 0.001).

We computed the β-NTI in soils across various vegetation types to assess the relative influences of stochastic and deterministic processes on community assembly. Homogeneous deterministic selection played a controlling role in the bacterial community structure (Figure 3c). The regression analysis based on the Bray–Curtis distance revealed a significant distance–decay relationship between Bray–Curtis similarity and spatial distance within the bacterial community in the Saihanba area (*R*^2^ = 0.25, *p* < 0.001). Upon converting geographical coordinate distances, the four vegetation types (comprising a total of 60 sampling points) yielded 1770 pairs of Bray–Curtis distances, with seven main distributions ranging from 0.019 km to 14.857 km (Figure 3d).

Correlation analysis results indicated that Shannon and Chao1 indices were significantly correlated with soil chemical properties, and the beta-diversity of bacterial communities (PC1, PC2) were significantly affected by SOM-C/N (Table 3). Multiple stepwise regression revealed that SOM-C/N, AP, and CEC were significant factors affecting bacterial alpha diversity, with SOM-C/N predominantly influencing the Shannon diversity index (Table 4).

### 3.3. Bacterial Community Composition and Influencing Factors

After processing downstream data using QIIME2 (version 2021.2), a total of 1,128,457 high-quality bacterial sequences were obtained, standardized at a sampling depth of 11,792 (minimum frequency). These sequences represented 26 phyla, 68 classes, 137 orders, 194 families, 285 genera, and 6528 OTUs.

**Figure 4:**
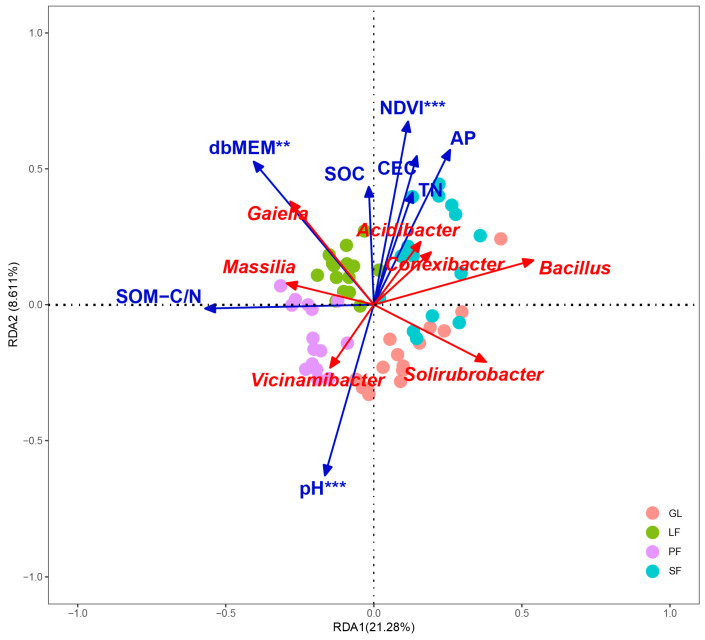
Redundancy analysis (RDA) of bacterial communities at the dominant genus and environmental variables. Red lines and arrows indicated dominant genera, and blue color represented environmental factors. ** *p* < 0.01, *** *p* < 0.001.

Bacteria with a relative abundance exceeding 1% were classified as dominant bacteria, while those with a relative abundance lower than 1%, along with uncultured bacteria, were categorized as others. The relative abundances of dominant bacteria exhibited notable variations across the four vegetation types (Kruskal–Wallis tests, *p* < 0.05, Figure S1, Tables S1–S5). At the genus level (Figure S1e–f), *Solirubrobacter* was the most widely distributed genus across all vegetation types (31.86% of GL, 30.73% of SF, 26.15% of PF, and 24.11% of LF). LF had the highest relative abundances of *Massilia* and *Gaiella*. *Acidibacter* was most abundant in SF. PF was rich in *Vicinamibacter*, while the abundance of *Bacillus* and *Conexibacter* in PF was the lowest compared to other vegetation types. Through RDA between dominant genera and environmental factors, RDA1 and RDA2 explained 21.28% and 8.61% of the variance information, respectively, with the two axes capturing 29.89% of the variance (Figure 4). The Mantel test results revealed that pH (*p* < 0.001), dbMEM (*p* < 0.01), and NDVI (*p* < 0.001) were identified as the primary environmental factors influencing bacterial communities (Table 5).

### 3.4. Functional Prediction of Bacterial Community in the Saihanba Area

Functional annotation of prokaryotic taxa (FAPROTAX) was employed to annotate potential functions of soil microbial communities in both grassland and plantations. A total of 1034 OTUs were annotated to 43 ecological functions, encompassing chemoheterotrophy (accounting for 43.07% abundance of the communities), aerobic chemoheterotrophy (40.25%), ureolysis (3.27%), predatory or exoparasitic (3.22%), fermentation (2.40%), and intracellular parasites (1.90%).

**Figure 5:**
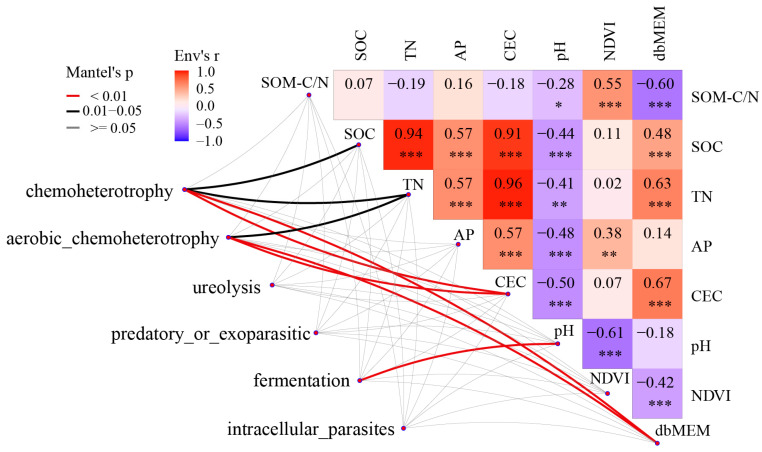
Network heat map between dominant functional groups and environmental factors. The connecting lines’ colors represent the significance level of functional groups concerning environmental factors. The grid colors and numbers depict the correlation magnitude between environmental factors. * *p* < 0.05, ** *p* < 0.01, *** *p* < 0.001.

The correlation heat map illustrates significant associations between dominant functional groups (relative abundance > 1%) and environmental factors (Figure 5). Chemoheterotrophy and aerobic chemoheterotrophy exhibited positive correlations with TN (r = 0.15 and r = 0.14, respectively), CEC (r = 0.24 and r = 0.23, respectively), and dbMEM (r = 0.71 and r = 0.72, respectively). Furthermore, chemoheterotrophy demonstrated a positive correlation with SOC (r = 0.12), while fermentation exhibited a positive correlation with pH (r = 0.26).

### 3.5. Co-Occurrence Network Analysis of Bacterial Communities

**Figure 6:**
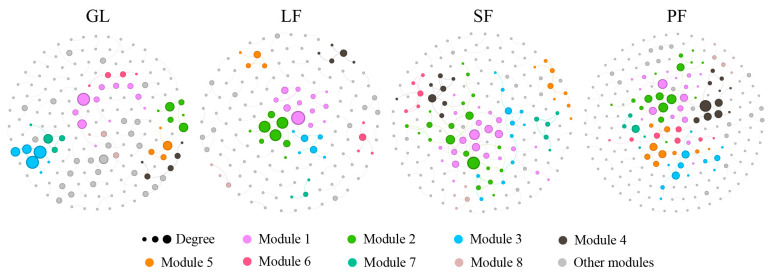
Co-occurrence networks of bacterial OTUs in four vegetations types (GL, grassland; LF, *L. gmelinii* forest; SF, *P. asperata* forest; PF, *P. sylvestris* forest). Nodes are colored based on modularity class, and sizes reflect the degree of connection. Edge thickness represents Spearman (*R* > |0.6|) and significant (*p* < 0.05) correlations.

Co-occurrence network analysis at the OTU level revealed altered topological properties among the four vegetation types (Figure 6, Table 6). The average degree, average path length and network diameter in the soil bacterial network structure of the plantation vegetation were higher than those of the grassland. Specifically, the SF exhibited the highest average degree at 1.874, while the PF had the largest average path length (4.267) and network diameter (11.913). Despite LF having fewer nodes and edges, the average degree of the soil network in plantation forest vegetation surpassed that of grassland, indicating a more complex bacterial network following afforestation. The grassland displayed the highest clustering coefficient (0.357) and modularity index (0.969). The modularity indices of the four types of vegetation are all greater than 0.4, indicating that the constructed networks have formed a good modular structure.

Additionally, the combination of degree and PageRank identified key species in the co-occurrence network (Table 7). GL, LF, SF, and PF each revealed 3, 4, 3, and 3 key OTUs, respectively. These taxa span five bacterial phyla, including Actinobacteriota, Acidobacteriota, Pseudomonadota, Verrucomicrobiota, and Methylomirabilota. At the class level, key OTUs in GL belonged to Vicinamibacteria and Chlamydiae. In plantation vegetation, key groups of soil bacteria were classified as Thermoleophilia, Methylomirabilia (exclusive to LF), and Gammaproteobacteria (unique to SF). The key species in the soil bacterial network of plantations are mainly dominant species, but the relative abundance of Chlamydiae in grassland is less than 1%.

## 4. Discussion

### 4.1. Influence of Soil Chemical Properties on Bacterial Alpha Diversity Post-Afforestation

Significant variations exist in soil conditions among different plantation tree species. Compared with LF and SF, PF exhibits a higher pH value (Table 1), consistent with studies on diverse vegetation types in semi-arid regions [39]. This distinction could stem from the increased acidity of surface litter in Pinus sylvestris forests [40]. We observed higher soil nutrient levels in LF compared to other stands. Prior research has also demonstrated the considerable impact of tree species on soil properties [41]. In our study, the C/N ratios of plantations followed the order PF > SF > LF, aligning with previous findings that underscore the potential influence of different forest types on soil carbon and nitrogen ratios [42]. In conclusion, distinct disparities exist in soil properties following afforestation with various tree species within the same area.

The impact of afforestation on soil bacterial alpha diversity was significant, being more than that of grassland (Figure 2), consistent with prior research findings [39]. PF showed significantly higher soil bacterial α-diversity, possibly due to increased soil SOM-C/N levels. The SOM-C/N ratio in PF significantly exceeded that of other vegetation types (Table 2), with multiple linear regression analysis confirming its role as the primary environmental factor influencing diversity shifts. Hence, we suggest that cultivating *Pinus sylvestris* forests enhances soil C/N, thereby fostering increased bacterial diversity. The plantation forest exhibited a moderate increase in alpha diversity compared to grassland, partially confirming our hypothesis. Furthermore, bacterial alpha diversity is notably influenced by soil chemical properties, including soil organic carbon (SOC), total nitrogen (TN), and available phosphorus (AP). Our previous study demonstrated that at broader spatial scales, temperature and precipitation primarily drive variations in soil microbial communities within *Pinus sylvestris* forest [28]. Notably, bacterial community composition is predominantly influenced by chemical properties, including total nitrogen, soil organic carbon, and pH levels. Conversely, soil microbial alpha diversity is impacted by soil physical attributes, such as salinity and moisture content [43,44]. Future research should more comprehensively address the effects of soil microclimate and physical factors like bulk density and moisture on microorganisms.

### 4.2. Afforestation’s Impact on Bacterial Community Structure and Assembly Processes

Significant differences in bacterial community structure emerged among the four vegetation types. The overlap of bacterial communities between plantations and grassland is only 8.39% in terms of shared OTUs, with plantations sharing less than 5% of OTUs. While the bacterial structure of soil in SF and PF partially overlapped with grassland vegetation, LF’s bacterial structure exhibited significant separation from other vegetation types. The SOC, TN, and CEC were significantly higher in LF compared to other vegetation types, likely contributing to the distinct separation of bacterial communities. However, our analysis revealed only a 36.8% variation in community structure, suggesting the influence of additional factors on bacterial community structure. Prior studies have underscored the significant impact of forest type on microbial community composition [45]. Notably, variations in understory vegetation can drive changes in litter production and soil bacterial composition [46]. Furthermore, mycorrhizal fungi, integral members of terrestrial ecosystems, occupy diverse ecological niches and perform various physiological and ecological roles [47,48,49]. Investigating the interactions between mycorrhizal fungi and bacteria within the soil ecosystem may unveil novel insights into the dynamics of microbial communities. Additionally, exploring how environmental factors modulate these interactions and their subsequent effects on ecosystem functioning would contribute to a more comprehensive understanding of belowground microbial ecology.

The assembly process of soil bacteria in plantation forests remained largely unchanged compared to grassland, with deterministic homogeneous selection exerting predominant influence. Previous studies have underscored the remarkable adaptability of bacterial communities to disturbances such as shifts in vegetation, potentially contributing to increased biome similarity [50]. Given that all four vegetation types in our study are characterized by aeolian sandy soil, it is plausible that homogeneous selection processes dominate bacterial interactions, reflecting adaptation to similar abiotic environments. Moreover, our analysis revealed a negative correlation between bacterial community similarity and geographic distance (Figure 3d), indicating distinct compositions of bacterial communities across sites with significant spatial disparities. However, the results of Variation Partitioning Analysis (VPA) suggest that soil properties exert a more pronounced influence on bacterial community compositions compared to geographic distance (Figure S2). Notably, SOM-C/N emerged as a key chemical property significantly impacting the dissimilarity of soil bacterial communities (PC1, PC2) (Table 3). Therefore, it appears that soil properties exhibit considerable variation among different vegetation types and play a pivotal role in shaping soil microbial structure.

### 4.3. Bacterial Community Composition and Functional Group Dynamics Post-Afforestation

Afforestation significantly changed the composition of soil bacteria at the genus level compared with grassland. In LF, *Gaiella* emerged as the prevailing soil bacterial genus, actively contributing to the nitrogen cycle and showcasing a sensitivity to vegetation and environmental factors [51]. SF soil hosted *Acidibacter*, *Conexibacter*, and *Bacillus* as the dominant genera, with *Acidibacter* displaying an affinity for soil organic matter and nitrogen compounds, suggesting a healthier soil condition [52]. Species of the genus *Conexibacter* can utilize carbohydrate hydrolysis as the primary source of energy and carbon, while also performing the reduction of nitrate to nitrite [53,54,55]. These characteristics suggest their potential contribution to soil carbon and nitrogen cycling. *Bacillus subtilis*, a bacterium commonly found in soil, has gained attention in recent years for its applications in seed protection and biological control [56]. With its renowned stress resistance, *Bacillus* could potentially be utilized in future forest management practices to promote the growth of trees. The dominant bacterial genus in the PF soil is *Vicinamibacter*, which has been identified as a significant species in the co-occurrence network of karst rocky desertification areas [57]. Certain members of this genus and its family exhibit potential chitin-degrading genes [58]. Therefore, *Vicinamibacter* species may serve a pivotal role in preserving community function. The RDA results underscored the strong impact of pH, SOM-C/N, and dbMEM on the bacterial community, aligning with prior study [59]. The decrease in soil pH after afforestation, coupled with a significant increase in SOM-C/N, suggested accelerated organic matter accumulation and improved soil environmental conditions [60]. TN content, primarily influenced by tree species, varied among vegetation types, demanding further exploration of microorganism-nitrogen interactions.

FAPROTAX is a functional annotation database compiled from current literature on cultivable bacteria. It contains over 7600 species across more than 80 functional groups (such as fermentation type, nitrate respiration type, nitrogen fixation type, aerobic chemoheterotrophic type, etc.) [37]. While it may not capture the functional phenotypes of all taxa within a community, FAPROTAX serves as a valuable tool for rapidly screening or grouping 16S bacterial data. This predictive approach has been widely utilized in assessing the ecological functions of soil bacterial communities in forest and grassland ecosystems [39,61]. Our functional prediction analysis of soil bacterial communities across different vegetation types in the Saihanba area revealed chemoheterotrophy and aerobic chemoheterotrophy as the dominant functional groups, correlating significantly with TN and SOC (*p* < 0.05). This indicates that shifts in soil carbon and nitrogen pools may predominantly influence the functional groups of soil bacteria. This finding aligns with Liang et al. [62], who demonstrated a relationship between chemoheterotrophic and aerobic chemoheterotrophic bacterial functional groups and the soil carbon cycle. Soil environmental conditions exert a strong influence on the distribution of these groups. In addition, fermentation showed a significant positive correlation with soil pH (*p* < 0.05) in our study, suggesting that soil pH could enhance the abundance and diversity of fermentative bacterial functional groups. Ureolysis participates in the soil nitrogen cycle (nitrogen fixation, nitrification, denitrification, etc.) process [63]. Intracellular parasites rely on host cells to grow and reproduce. The research conducted by Zhao et al. [64] indicates that long-term nitrogen application to soil alters pH levels and reduces the abundance of predatory or exoparasitic bacteria. The primary OTUs identified with predatory or exoparasitic functions include OTU114, OTU136, and OTU195, all belonging to Myxococcales. Meanwhile, the main OTUs identified with intracellular parasites include OTU578, OTU754, and OTU780, all belonging to Chlamydiales. However, ureolysis, predatory or exoparasitic, and intracellular parasites are not related to environmental factors in this study. There may be potential soil factors such as texture that have not been taken into account. Further research should investigate these aspects to obtain a comprehensive understanding.

### 4.4. Increased Complexity of Bacterial Co-Occurrence Networks Post-Afforestation

The complexity of the soil bacterial network, as measured through the number of nodes, edges, and average degree, followed the order SF > PF > LF > GL, indicating an enhanced network complexity after afforestation, consistent with prior research [65]. Previous studies have highlighted the importance of soil properties in driving microbial network structure [66,67]. Besides soil properties and vegetation characteristics, microbial alpha diversity may also regulate microbial networks. In this study, the alpha diversity of soil bacteria in the PF was significantly higher than that of other vegetation types. The increased complexity of the PF network structure may stem from its higher community alpha diversity. Both the number of nodes and edges in the SF and PF were significantly higher than those in GL, suggesting that changes in aboveground vegetation enhanced soil–bacterial interaction. The soil microbial networks of all four vegetation types exhibited a modular structure, where species within the same module may share similar ecological functions or niches, thereby maintaining community stability [68,69]. Moreover, the Chlamydiae identified in GL, although not dominant taxa, may harbor more unique OTUs that complement the functions provided by other dominant species [70]. Research indicates that Chlamydia is significantly enriched in acidic and nutrient-poor soils [71], suggesting that grassland soil is poorer in nutrients than plantation soil, with afforestation improving soil environmental conditions to some extent. Despite a relatively low average degree of co-occurrence network for all four vegetation types, possibly due to data limitations, the general trend indicated more intricate soil bacterial co-occurrence networks in planted forests compared to grassland. This underscores the significant contribution of afforestation to the complexity and resilience of soil bacterial communities, further emphasizing its critical role in ecological restoration endeavors.

## 5. Conclusions

Based on our study in the Saihanba region, soil bacterial communities in artificially afforested LF, SF, and PF exhibit significant differences in composition and diversity compared to GL. In line with our hypothesis, the afforestation process significantly enhanced soil bacterial alpha diversity, closely linked to changes in soil chemical properties particularly with an elevation in AP, CEC, and SOM-C/N. Notably, soil properties exerted a stronger influence on bacterial community structure than geographic distance, with SOM-C/N emerging as a significant factor affecting the dissimilarity of soil bacterial communities. Furthermore, afforestation led to increased complexity in soil bacterial co-occurrence networks, indicating heightened microbial interactions. These findings provide valuable insights into the ecological impacts of afforestation and highlight its critical role in shaping soil microbial dynamics. Further investigations into the mechanisms governing plant–microbe interactions and their implications for ecosystem functioning will be essential for refining afforestation strategies and advancing our understanding of microbial ecology in terrestrial ecosystems.

## Figures and Tables

**Table 1:** Basic information of four sampling sites in the Saihanba area.

Vegetation	GL	LF	SF	PF
Geographical coordinates	42°21′35″ N, 117°13′2″ E	42°21′32″ N, 117°23′39″ E	42°24′17″ N, 117°16′53″ E	42°24′14″ N, 117°15′31″ E
Stand age (a)	-	39	38	36
Altitude (m)	1439.7	1724.2	1528.5	1522.2
Average DBH (cm)	-	29.32 ± 2.93	20.60 ± 4.08	28.21 ± 2.43
Soil type	aeolian sandy soil	aeolian sandy soil	aeolian sandy soil	aeolian sandy soil

**Table 2:** Soil properties of four vegetation types in the Saihanba area (mean values ± standard error).

Soil Properties	GL	LF	SF	PF
pH	6.68 ± 0.03 ^a^	5.97 ± 0.04 ^b^	5.90 ± 0.22 ^b^	6.38 ± 0.03 ^a^
SOC (g/kg)	13.10 ± 1.66 ^b^	20.16 ± 1.55 ^a^	14.66 ± 0.89 ^b^	12.16 ± 0.86 ^b^
TN (g/kg)	1.30 ± 0.15 ^b^	1.87 ± 0.12 ^a^	1.29 ± 0.07 ^b^	0.93 ± 0.07 ^c^
SOM-C/N	9.92 ± 0.17 ^d^	10.72 ± 0.16 ^c^	11.35 ± 0.21 ^b^	13.24 ± 0.28 ^a^
AP (mg/kg)	3.78 ± 0.20 ^b^	4.52 ± 0.27 ^a^	4.81 ± 0.19 ^a^	3.80 ± 0.14 ^b^
CEC (cmol(+)/kg)	6.80 ± 0.87 ^b^	11.94 ± 0.85 ^a^	7.68 ± 0.34 ^b^	4.91 ± 0.39 ^c^

One-way ANOVA was performed using Duncan’s method, with different letters in the same row indicating significant differences (*p* < 0.05, n = 15).

**Table 3:** Spearman correlation coefficient between bacterial diversity and soil properties.

Diversity Indices	pH	TN	AP	CEC	SOC	SOM-C/N
Shannon	0.297 *	−0.323 *	−0.361 **	−0.359 **	−0.234	0.313 *
Chao1	0.398 **	−0.520 ***	−0.311 *	−0.568 ***	−0.425 ***	0.237
Pielou	0.006	0.084	−0.206	0.099	0.109	0.189
PC1	−0.159	0.213	0.225	0.233	0.127	−0.321 *
PC2	0.042	−0.075	−0.188	−0.061	0.060	0.436 ***

PC1 and PC2 represent the principal coordinates in the principal co-ordinate analysis (PCoA) utilizing Bray–Curtis distance. pH denotes the pH value; SOC stands for soil organic carbon (g kg^−1^); AP signifies available phosphorus (mg kg^−1^); TN refers to total nitrogen (g kg^−1^); CEC represents cation exchange capacity (cmol(+) kg^−1^). Significance levels: * *p* < 0.05; ** *p* < 0.01; *** *p* < 0.001.

**Table 4:** Linear stepwise regression analysis between soil chemical properties and bacterial alpha diversity.

Alpha Index	Variable	Slope	Std. Error	t Value	Pr(>|t|)	Independent Contribution (%)	*R* ^2^	*p*
Shannon	AP	−0.08	0.03	−2.93	<0.01	37.8	0.27	<0.001
	SOM-C/N	0.08	0.02	4.76	<0.001	62.2		
Chao1	CEC	−15.78	7.34	−2.15	<0.05	34.78	0.43	<0.001
	AP	−35.78	12.34	−2.90	<0.01	30.50		
	SOM-C/N	15.43	7.06	2.18	<0.05	34.68		
Pielou	CEC	0.003	0.001	2.99	<0.001	38.35	0.19	<0.01
	AP	−0.01	0.005	−2.28	<0.005	17.22		
	SOM-C/N	0.007	0.002	2.95	<0.001	44.64		

**Table 5:** Mantel test between dominant genera of bacterial communities and environmental factors.

	pH	TN	SOC	AP	CEC	SOM-C/N	dbMEM	NDVI	Total
*r*	0.230	−0.057	−0.066	0.012	−0.078	0.003	0.123	0.271	−0.07
*p*	0.001 ***	0.857	0.915	0.385	0.948	0.433	0.005 **	0.001 ***	0.908

The envfit function was used to test the significance of each soil properties in RDA (** *p* < 0.01; *** *p* < 0.001).

**Table 6:** Topological properties of bacterial co-occurrence networks in the Saihanba area.

Vegetation	Nodes	Edges	Average Degree	Average Path Length	Network Diameter	Network Density	Clustering Coefficient	Modularity
GL	165	116	1.406	1.600	5.945	0.009	0.357	0.969
LF	144	102	1.417	2.838	6.845	0.010	0.340	0.932
SF	175	164	1.874	3.582	8.505	0.011	0.260	0.835
PF	207	163	1.575	4.267	11.913	0.008	0.273	0.924

**Table 7:** Bacterial keystone taxa across four vegetation types.

Vegetation	OTU ID	Phylum	Class	Degree	PageRank	Modularity Class
GL	OTU175	Acidobacteriota	Vicinamibacteria	4	0.011	1
GL	OTU351	Acidobacteriota	Vicinamibacteria	4	0.009	2
GL	OTU966	Verrucomicrobiota	Chlamydiae	4	0.009	2
LF	OTU31	Actinobacteriota	Thermoleophilia	6	0.015	1
LF	OTU36	Actinobacteriota	Thermoleophilia	5	0.011	2
LF	OTU99	Acidobacteriota	Vicinamibacteria	5	0.011	2
LF	OTU40	Methylomirabilota	Methylomirabilia	5	0.011	2
SF	OTU8	Actinobacteriota	Thermoleophilia	10	0.016	1
SF	OTU53	Pseudomonadota	Gammaproteobacteria	8	0.013	1
SF	OTU111	Acidobacteriota	Vicinamibacteria	7	0.012	2
PF	OTU8	Actinobacteriota	Thermoleophilia	6	0.010	4
PF	OTU576	Acidobacteriota	Vicinamibacteria	5	0.009	1
PF	OTU411	Acidobacteriota	Vicinamibacteria	5	0.009	1

## Data Availability

Data are contained within the article.

## Supplementary Materials

The following supporting information can be downloaded at: https://www.mdpi.com/article/10.3390/microorganisms12030479/s1, Figure S1: Community composition and differential analysis of soil bacteria in the Saihanba region. (a–e) Relative abundance of dominant bacteria from phyla to genera among four vegetation types. (f) LEfSe analysis of genera between vegetation types (LDA significance threshold > 2.0). * *p* < 0.05, *** *p* < 0.001. Figure S2: The relative importance of geographical distance and soil properties in shaping bacterial community compositions. Table S1: Abundance and Kruskal–Wallis tests of bacterial phyla in four vegetation types in the Saihanba region (Please refer to the excel file). Table S2: Abundance and Kruskal–Wallis tests of bacterial classes in four vegetation types in the Saihanba region (Please refer to the excel file). Table S3: Abundance and Kruskal–Wallis tests of bacterial orders in four vegetation types in the Saihanba region (Please refer to the excel file). Table S4: Abundance and Kruskal–Wallis tests of bacterial families in four vegetation types in the Saihanba region (Please refer to the excel file). Table S5: Abundance and Kruskal–Wallis tests of bacterial genera in four vegetation types in the Saihanba region (Please refer to the excel file).
