# Supplementary material for: Afforestation-Induced Shifts in Soil Bacterial Diversity and Community Structure in the Saihanba Region
Source: Microorganisms. 2024 Feb 27;12(3):479. doi: 10.3390/microorganisms12030479 (PMC10974129; doi:10.3390/microorganisms12030479)
Supplement: Supplementary file 1 [file microorganisms-12-00479-s001.zip › Supplementary materials.pdf]

Supplementary Materials

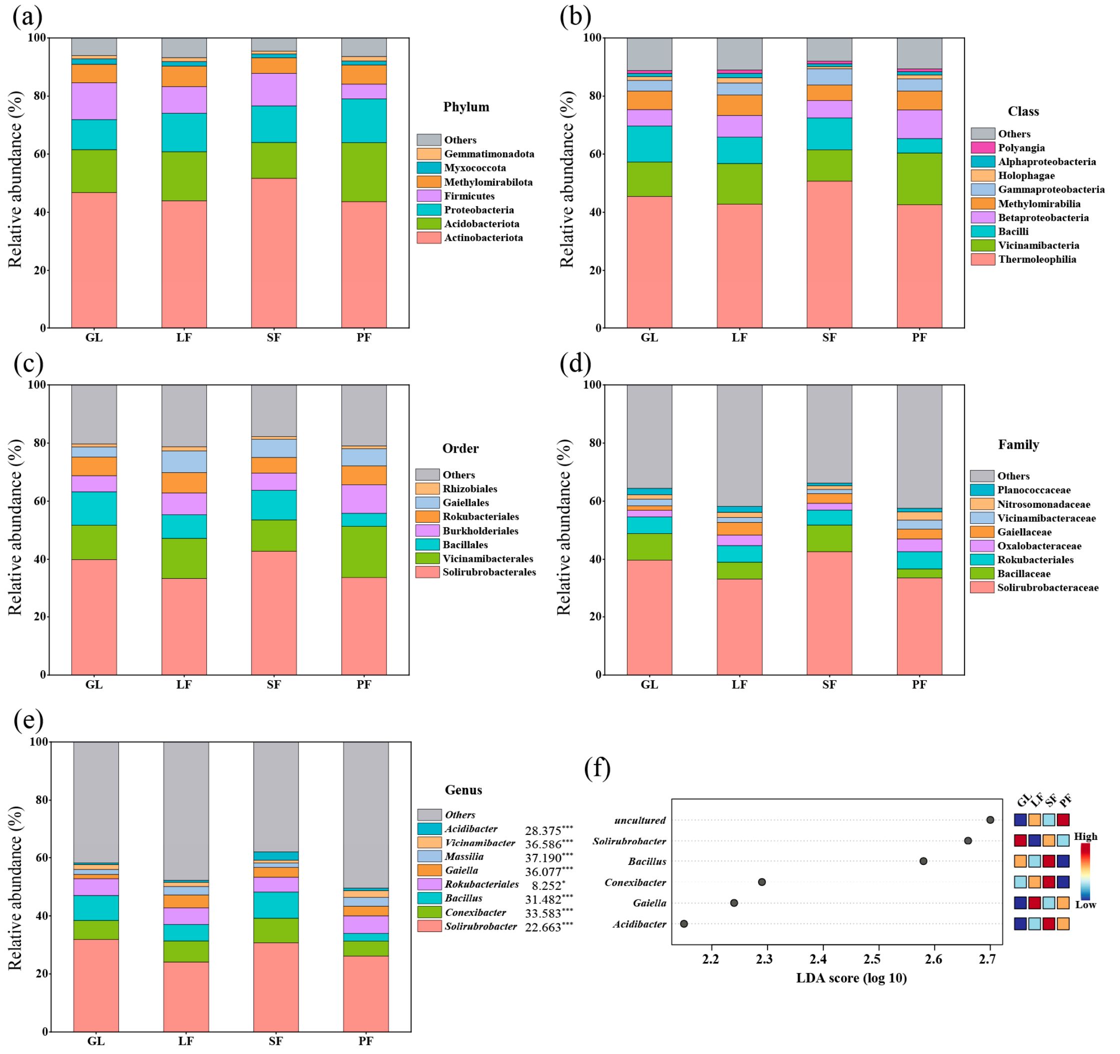

Figure S1. Community composition and differential analysis of soil bacteria in the Saihanba region. (a-e) Relative abundance of dominant bacteria from phyla to genera among four vegetation types. (f) LEfSe analysis of genera between vegetation types (LDA significance threshold > 2.0). *$p < 0.05$, ***$p < 0.001$.

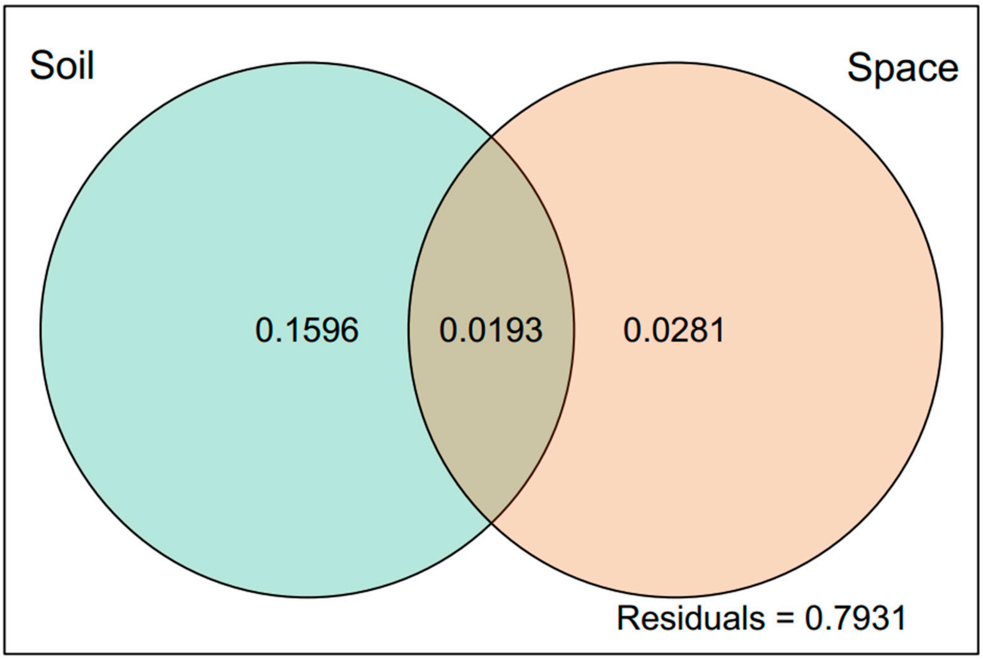

Figure S2. The relative importance of geographical distance and soil properties in shaping bacterial community compositions.

Table S1. Abundance and Kruskal–Wallis tests of bacterial phyla in four vegetation types in the Saihanba region (Please refer to the excel file).

Table S2. Abundance and Kruskal–Wallis tests of bacterial classes in four vegetation types in the Saihanba region (Please refer to the excel file).

Table S3. Abundance and Kruskal–Wallis tests of bacterial orders in four vegetation types in the Saihanba region (Please refer to the excel file).

Table S4. Abundance and Kruskal–Wallis tests of bacterial families in four vegetation types in the Saihanba region (Please refer to the excel file).

Table S5. Abundance and Kruskal–Wallis tests of bacterial genera in four vegetation types in the Saihanba region (Please refer to the excel file).
